# Detection and Analysis of Genes Affecting the Number of Thoracic Vertebrae in Licha Black Pigs

**DOI:** 10.3390/genes15040477

**Published:** 2024-04-10

**Authors:** Yuan Wang, Min Wang, Xiaojin He, Ruilan Dong, Hongjiang Liu, Guanghui Yu

**Affiliations:** 1 College of Animal Science and Technology, Qingdao Agricultural University, Qingdao 266109, China; wangyuan@qau.edu.cn (Y.W.); min@qau.edu.cn (M.W.); hexiaojin2021@163.com (X.H.); dongrl@qau.edu.cn (R.D.); 2 Bureau of Agriculture and Rural Affairs of Jiaozhou, Jiaozhou 266300, China; jzpjdjz@163.com

**Keywords:** Licha black pig, number of thoracic vertebrae, selection signal regions, functional genes

## Abstract

The number of thoracic vertebrae (NTV) in pigs is an important economic trait that significantly influences pork production. While the Licha black pig is a well-known Chinese pig breed with multiple thoracic vertebrae, the genetic mechanism is still unknown. Here, we performed a selective signal analysis on the genome of Licha black pigs, comparing individuals with 15 NTV versus those with 16 NTV to better understand functional genes associated with NTV. A total of 2265 selection signal regions were detected across the genome, including 527 genes and 1073 QTL that overlapped with the selection signal regions. Functional enrichment analysis revealed that *LRP5* and *SP5* genes were involved in biological processes such as bone morphogenesis and Wnt protein binding. Furthermore, three genes, *LRP8*, *DEF6*, and *SCUBE3*, associated with osteoblast differentiation and bone formation, were located within or close to the QTL related to bone development and vertebrae number. These five genes were hypothesized to be potential candidates for regulating the NTV trait in Licha black pigs. Our findings revealed several candidate genes that play crucial roles in NTV regulation and provide a theoretical foundation to understand the genetic mechanism of the NTV trait in pig breeding.

## 1. Introduction

The number of vertebrae in pigs is an economically important trait, composed of cervical vertebrae, thoracic vertebrae, lumbar vertebrae, sacral bone, and caudal vertebrae. The number of thoracic vertebrae (NTV) varies from 13 to 17 and is equivalent to the number of ribs, potentially influencing carcass length and body weight [1,2]. Therefore, in the pig industry, selecting multi-vertebral pigs by molecular breeding means can increase pork production and economic benefits.

China has a wealth of pig genetic resources, accounting for roughly one-third of the world’s total pig breeds [3]. Numerous indigenous pig breeds in China display distinctive traits, including disease resistance, efficient feed conversion, and high fertility [4]. The preservation and utilization of these indigenous pig breeds are essential for maintaining biodiversity and for the development of new pig breeds with improved traits. The Licha black pig is a popular local pig breed, primarily found in the Jiaodong Peninsula of Shandong Province, China. It is worth mentioning that Licha black pig is distinguished from other local breeds by a multi-vertebral trait manifested by one additional thoracic vertebrae [5].

NTV has a moderate heritability of approximately 0.6 [1,6]. Previous research has demonstrated that NTV is a polygenic trait influenced by multiple quantitative trait loci (QTL). Yang et al. [7] discovered that the *VRTN* gene located on Sus scrofa chromosome (SSC) 7 was significantly associated with NTV in the Duroc, Landrace, and White Duroc × Erhualian F2 population, and the *VRTN* mutation site potentially also influenced carcass length and teat number. Niu et al. [8] revealed that the *GREB1L* and *MIB1* genes on SSC6 and the *ABCD4* gene on SSC7 were related to NTV in the Beijing black pig population. Furthermore, the *FOS* and *BMPR1A* genes, located on SSC7 and SSC14, respectively, were identified as the candidate genes regulating NTV in a Large White × Minzhu intercross pig population [9]. As previously stated, somite, formed from the presomitic mesoderm during early embryogenesis, regulates the number of vertebrae [10,11]. Moreover, research evidence on model organisms revealed that the Notch, Wnt, and Retinoic acid signal pathways can regulate somite development to ensure normal formation and differentiation via segmentation clock regulation [12]. Among them, the Wnt signaling pathway is critically linked to various developmental processes, including gastrulation, organ development, and tissue homeostasis. In particular, during vertebrae development, the Wnt signaling pathway influences the development of somites, some of which develop into vertebrae [10].

While candidate genes for NTV have previously been identified, the studies primarily focused on Western pig breeds and hybrid bred from Western and Chinese pig breeds. On the contrary, the Licha black pig is an indigenous breed formed by long-term breeding of local residents, which is less impacted by modern hybrid breeding and has less introgression of Western pig lineage. The purpose of the current study was to identify candidate genes that play crucial roles in NTV regulation and to provide a theoretical foundation for the increase of NTV in Licha black pigs.

## 2. Materials and Methods

### 2.1. Animal and Sample Collection

One-month-old Licha black pigs for this study were selected from the National Nucleus Licha Black Pig Conservation Farm in Jiaozhou, Shandong Province, China. DUAL VET X-Plus (Sedecal, Madrid, Spain), a portable X-ray machine, was utilized to take an X-ray picture of each individual, and the number of thoracic vertebrae was accurately counted by the staff. A total of 19 Licha black pigs, including 9 with 16 NTV and 10 with 15 NTV, were selected for the subsequent analysis. We strictly followed the Animal Care and Use of Qingdao Agricultural University (Qingdao, China) for the relevant experimental procedures. Ear tissue samples were collected using scissors and placed in an anhydrous ethanol-containing centrifuge tube. All the tools and equipment used for sample collection were sterilized by heat or ultraviolet rays.

### 2.2. Whole Genome Resequencing

Genomic DNA from ear tissues was extracted using TIANamp Genomic DNA kits (Tiangen Biotech, Beijing, China). The concentration and purity of genomic DNA were detected using a NanoDrop™ 2000 (Thermo Fisher, Waltham, MA, USA). DNA samples with a light absorption ratio (A260/280) between 1.8 and 2.0 and a concentration > 50 ng/μL were used in the subsequent steps. DNA libraries were constructed for each individual using an MGIEasy FS DNA Prep kit (BGI, Shenzhen, China) following the manufacturer’s instructions. Paired-end sequencing using the MGISEQ-2000 platform (BGI, Shenzhen, China) yielded 150 bp-sized sequencing reads.

### 2.3. Quality Control and Reads Mapping

To ensure the reliability of bioinformatics analysis, the NGS QC Toolkit [13] was used to remove low-quality paired reads of the sequencing data. Reads containing greater than 5% unidentified nucleotides (N) longer than 50% bases with phred quality less than 5 were eliminated. BWA 0.7.17 software [14] was used to map clean data to the reference genome Sus scrofa 11.1 (https://www.ensembl.org/Sus_scrofa/Info/Index, accessed on 1 January 2023). SAMtools 1.12 software [15] was employed to perform local realignment and eliminate PCR duplicates. Subsequently, SNP for each individual was detected using the GATK 4.2.0 software [16], and the genotype data were quality controlled using Plink 1.90 software [17]. Individuals with genotyping call rates less than 90%, SNP with a call rate less than 90%, minor allele frequencies less than 0.01, Hardy–Weinberg equilibrium *p*-value less than 1 × 10^−6^, and SNP on sex chromosomes were excluded.

### 2.4. Genome Scanning for Selective Signal Analysis

The whole genome selection signals were determined by comparing individuals with 15 NTV to those with 16 NTV. A selective scanning analysis was performed using VCFtools 0.1.16 software [18] and setting a 100 KB sliding window with a step size of 10 KB. The genetic differentiation value (F_ST_) between the two groups was calculated, with the empirical top 1% as the threshold (F_STtop0.01_ = 0.18). Genes partially or completely overlapping with selection signal regions were selected based on the Sus scrofa 11.1 genome assembly using the BioMart data management (http://www.biomart.org/, accessed on 1 January 2023). Furthermore, these selection signal regions were also compared with pig QTL from the animal QTL database (https://www.animalgenome.org/cgi-bin/QTLdb/, accessed on 1 January 2023).

### 2.5. Enrichment Analysis

To provide insight into the functional enrichment of genes, Gene Ontology (GO) and Kyoto Encyclopedia of Genes and Genomes (KEGG) pathway enrichment analysis of selected genes were performed using the KOBAS 3.0 software [19]. Fisher’s exact test was employed to determine the significance of the enriched terms and pathways [20], with *p*-value less than 0.05 deemed significant for enrichment analysis [21].

## 3. Results

### 3.1. Quality and Statistics of the Sequencing Data

The MGISEQ-2000 platform yielded 625.39 GB of raw sequencing data from 19 Licha black pigs, with each sample generating 30.55~36.39 GB of raw data. Quality screening yielded 616.94 GB of clean data. The average effective sequencing rate was 98.65%, with an average Q30 of 90.34%. Clean reads were mapped to the pig reference genome using the BWA 0.7.17 software, and the average mapping rate of the clean data was 99.09%. The average effective depth of the reference genome coverage was 12.58-fold, ranging from 11.33-fold in the MH-B4 sample to 13.95-fold in the MH-R12 sample. These findings showed that all sequence data generated in this study were suitable for subsequent analysis (Table S1).

### 3.2. Selective Signal Analysis 

**Figure 1:**
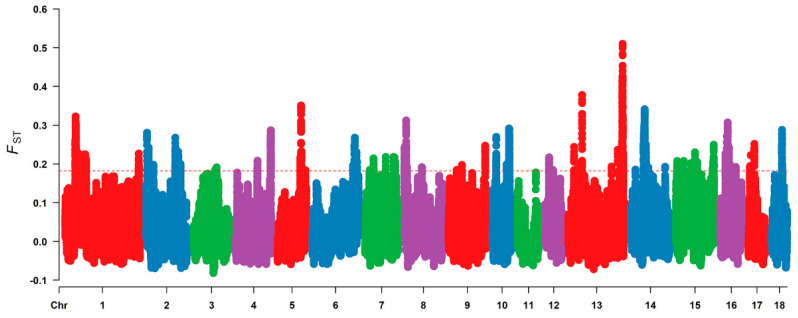
The Manhattan plot of selective signal analysis for the NTV. The F_ST_ distribution map was drawn along pig chromosomes from 1 to 18 (different chromosomes were separated by color). The red dashed line indicated the significance threshold, and the F_ST_ value was 0.18.

We applied several criteria to filter the sequence data; 19 individuals and 19,436,395 SNPs were retained for selective signal analysis. Through the analysis of the F_ST_ value (top 1%), a total of 2265 selection signal regions were distributed on the 17 autosomes of the pig genome (Figure 1), and these regions were listed in Table S2. In addition, the statistics of selection signal regions on the pig autosomes are shown in Table 1. Of these, SSC1 contained the greatest number of selection signal regions (380), while SSC3 had the least number of selection signal regions (4). The most significant region was located in the 199.28–199.38 MB of SSC13. Furthermore, 527 genes overlapping with selection signal regions were selected to perform enrichment analysis (Table S3), and Table 2 listed the top 20 selection signal regions that contained the overlapping genes.

### 3.3. Functional Enrichment Analysis

**Figure 2:**
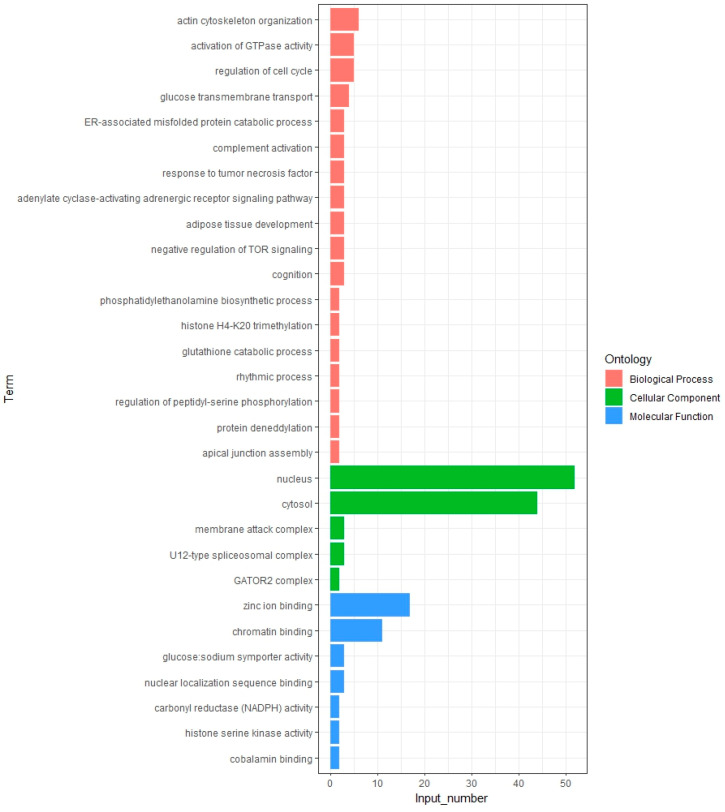
The top 30 enriched GO terms of the genes overlap with selection signal regions. The Y axis represents different GO terms. The X-axis represents the number of genes enriched in the GO term.

GO analysis showed that these genes were significantly enriched in 130 GO terms, including 78 GO terms in biological processes, 28 GO terms in cellular components, and 24 GO terms in molecular function (Table S4). The top 30 GO terms are outlined in Figure 2. These GO terms were primarily related to biological processes, including regulation of cell cycle, glucose transmembrane transport, adipose tissue development, regulation of microtubule cytoskeleton organization, positive regulation of MAPK cascade, and negative regulation of inflammatory response. We particularly focused on the terms highly likely related to bone development, including bone morphogenesis and Wnt protein binding, with *LRP5*, *SP5*, and *TRABD2B* genes implicated in these processes.

**Figure 3:**
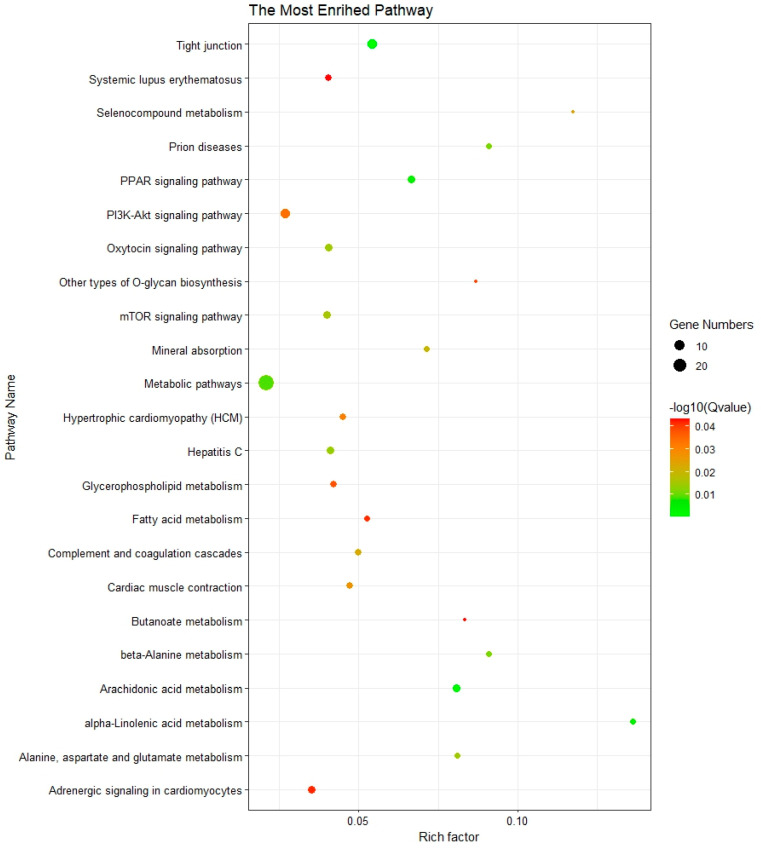
The significantly enriched KEGG pathways of the genes overlap with selection signal regions. The Y axis represents the function of enriched pathways. The X-axis represents the enrichment factor. The size of the dot indicates the number of genes enriched in the pathway, and the color corresponds to the different *p*-value ranges.

KEGG enrichment analysis revealed 25 significantly enriched pathways; further investigation of these pathways could reveal their biological relevance and potential significance (Figure 3, Table S5). These pathways were implicated in various regulatory pathways, including the PPAR signaling pathway, α-linolenic acid metabolism, glycerophospholipid metabolism, arachidonic acid metabolism, oxytocin signaling pathway, mTOR signaling pathway, and metabolic pathways. The involvement of these pathways suggests a complex interplay between lipid metabolism, cellular growth, and energy regulation.

### 3.4. Comparison of Selective Signal Regions and QTL Database

Compared with the QTL database in pigs, these selective signal regions harbored in or partially overlapping with 1073 QTL (Table S6), related to traits such as average backfat thickness, intramuscular fat content, corpus luteum number, teat number, body weight, bone mineral content, glucose level, white blood cell number, and front feet conformation. The presence of these regions highlights their potential as genetic markers in selective breeding programs. In this study, we focused on QTL associated with bone development and vertebra number, and 13 QTL, including bone mineral content, thoracic vertebra number, lumbar vertebra number, cervical vertebra length, cannon bone circumference, and spinal curvature, were identified (Table 3). Moreover, 68 genes overlapping with selection signal regions were also located within or close to these QTL (Table 3), with *LRP8*, *DEF6*, and *SCUBE3* genes playing critical roles in regulating osteoblast differentiation and bone formation.

## 4. Discussion

The Licha black pig is an important local pig breed in China, with qualities such as feed efficiency, disease resistance, and high fertility rate. In addition, the Licha black pig has one more NTV than other local pig breeds [22]. However, the genetic basis of the NTV in Licha black pig is yet unknown, which may limit the full exploitation of their genetic potential. In the present study, we employed F_ST_ tests to detect selection signal regions in the genome of Licha black pigs between individuals with 15 NTV and those with 16 NTV, which will help us to identify genes related to vertebrae development and improve our understanding of the genetic mechanisms of NTV trait in pig breeding.

A total of 527 genes overlapping with selection signal regions were subjected to functional enrichment analysis in the present investigation; we focused more on functional genes associated with somite formation and bone development. According to the results of functional enrichment analysis, *LRP5*, *SP5*, and *TRABD2B* genes were discovered to be involved in biological processes such as bone morphogenesis and Wnt protein binding; understanding their roles can provide insights into the molecular mechanisms of bone formation. The *LRP5* gene on SSC2 potentially plays a pivotal role in the processes of somite formation and bone development via the Wnt signaling pathway. This pathway is instrumental in the regulation of cell fate and differentiation during embryonic development, particularly in the formation of the axial skeleton. *LRP5* functional loss mutations can lead to a low bone mass phenotype, whereas *LRP5* functional gain mutations result in a dominant high bone mass phenotype [23]. Meanwhile, osteocyte-secreted sclerostin can serve as an endogenous Wnt signaling inhibitor by blocking the interaction of the Wnt ligand and *LRP5*, regulating bone mass and strength [24]. The *SP5* gene, located on SSC15, encodes a member of the SP family of Zinc-finger DNA binding proteins, which has been described as a Wnt/β-catenin target gene capable of acting upstream or within bone morphogenesis [25]. The role of the *SP5* gene in bone morphogenesis is multifaceted. It can control the balance between bone formation and resorption, which is essential for maintaining skeletal integrity. Huggin et al. [26] discovered that the *SP5* gene could induce the termination of a transcriptional program initiated by Wnt signaling, and this type of dampening gene expression is crucial for ensuring the completion of developmental processes. As a result, we hypothesize that both *LRP5* and *SP5* genes potentially may play a role in the regulation of NTV trait in Licha black pigs.

Moreover, we compared the selection signal regions with the pig QTL database, focusing on QTL related to bone development and vertebrae number. Of note, 68 genes overlapping with selection signal regions were found within or close to these QTLs, which aided in precise positioning and narrowing of the target area. Finally, three genes, *LRP8*, *DEF6*, and *SCUBE3*, were revealed as functional genes related to the NTV trait. The *LRP8* gene is located on SSC6, close to the QTL associated with thoracic vertebra number, and exerts a similar function to the *LPR5* gene, primarily playing a role in the formation and development of bones. Zhang et al. [27] discovered that *LRP8* could function as a positive regulator of the Wnt signaling pathway, promoting Wnt-induced osteoblast differentiation; this finding underscores the importance of *LRP8* in maintaining normal skeletal development. Moreover, *LRP8* knockout mice induce a defect in bone formation. Both *DEF6* and *SCUBE3* genes, located on SSC7, overlap with the QTL of cervical vertebra length. The *DEF6* gene, also known as *IBP* or *SLAT*, is first identified as an activator of Rho GTPases with distinct molecular structures [28]. Pei et al. [29] were the first to discover the association of *DEF6* with bone mineral density at different bone sites. Deng et al. [30] later investigated the role of *DEF6* in osteoblast differentiation and bone formation, and their results indicated that *DEF6* inhibited osteoblast differentiation and mineralization both in vitro and in vivo, and *DEF6* knockout mice displayed an osteoporotic phenotype with increased osteoclast formation. The *SCUBE3* gene encodes a member of the signal peptide family, which can function as co-receptors for various growth factors [31]. Bone morphogenetic protein (BMP), a growth factor, plays a critical role in bone formation and cartilage development. *SCUBE3* can modulate the BMP signaling pathway, enhancing the response and ensuring proper cellular differentiation and tissue formation. *SCUBE3* can also function as a BMP co-receptor, and its aberrant function of *SCUBE3* in mice impairs BMP-mediated chondrogenesis and ossification [32]. The regulation of *SCUBE3* is essential to prevent skeletal abnormalities and maintain the balance of bone and cartilage formation.

**Figure 4:**
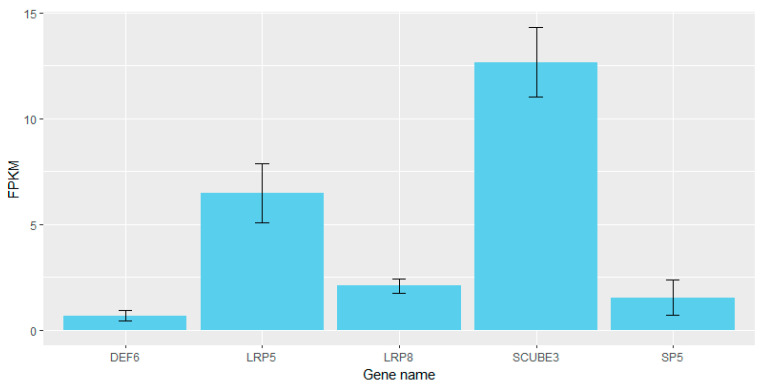
The FPKM of *LRP5*, *SP5*, *LRP8*, *DEF6*, and *SCUBE* genes in mice. The Y axis represents the value of FPKM. The X-axis represents the different gene names.

In addition, previous studies showed that the 7.5–9.5 days period of mouse gestation is a critical stage for the development of somite [8,33]. Therefore, based on the transcriptomic data of three mouse embryonic development at 9.5 days in a public database (https://figshare.com/s/496d0d17ad585717080c, accessed on 1 January 2023), we obtained the expression levels of the genes, expressed as the number of fragments per kilobase of exon per million mapped fragments (FPKM), and found that *LRP5*, *SP5*, *LRP8*, *DEF6* and *SCUBE* genes can be expressed during the critical stage of mouse somite development (Figure 4). Among them, the expression of the *SCUBE* gene was the highest, while the expression of *the DEF6* gene was the lowest. We further inferred that *LRP5*, *SP5*, *LRP8*, *DEF6*, and *SCUBE* genes are potential candidate genes associated with NTV traits in Licha black pigs.

## 5. Conclusions

This work discovered 2265 selective signal regions in the genome of the Licha black pig across distinct NTV. 5 functional genes associated with osteoblast differentiation and bone formation, namely, *LRP5*, *SP5*, *LRP8*, DF, and *SCUBE3*, were identified as key NTV candidates. Our findings provide new insights into the genetic basis of the NTV trait in Licha black pigs.

## Figures and Tables

**Table 1:** The statistic of selection signal regions on the pig autosomes.

SSC	Selection Signal Region Number	Length (Mb) ^1^	Autosome Coverage (%) ^2^	Gene Number ^3^
1	380	38	13.85	86
2	110	11	7.24	32
3	4	0.4	0.3	3
4	130	13	9.93	19
5	74	7.4	7.08	20
6	98	9.8	5.74	24
7	40	4	3.28	14
8	192	19.2	13.82	78
9	25	2.5	1.79	12
10	171	17.1	24.65	15
12	15	1.5	2.44	7
13	340	34	16.32	52
14	342	34.2	24.13	111
15	94	9.4	6.69	24
16	171	17.1	21.39	21
17	31	3.1	4.88	4
18	48	4.8	8.57	5
Total ^4^	2265	226.5	10	527

^1^ Represents the total length of the selection signal region on this autosome, and autosome length is expressed in Mb units; ^2^ represents the proportion of the total length of the selection signal region on this autosome to the total length of this autosome; ^3^ represents the number of genes overlapping with the selection signal region on this autosome; ^4^ represents the total number and length of selection signal regions, and total number of overlapping genes on the 17 autosomes.

**Table 2:** Top 20 selection signal regions overlapping with genes.

SSC	Bin Start (bp) ^1^	Bin Eed (bp)	F_ST_^2^	Gene Name ^3^
13	199,280,001	199,380,000	0.509862	*LOC106505853*, *LOC110256478*, *LOC106505851*
13	198,830,001	198,930,000	0.425367	*LOC106505851*
13	198,650,001	198,750,000	0.410486	*LOC106508030*
13	198,630,001	198,730,000	0.401655	*RUNX1*
13	50,480,001	50,580,000	0.377898	*ARL6IP5*, *LMOD3*, *LOC110256266*, *UBA3*
13	50,470,001	50,570,000	0.357269	*TMF1*
5	85,790,001	85,890,000	0.351059	*LOC102160458*
13	199,770,001	199,870,000	0.348404	*CBR3*, *DOPEY2*
14	49,210,001	49,310,000	0.34131	*BCR*
14	49,530,001	49,630,000	0.338639	*GGT1*, *GUCD1*, *LRRC75B*, *SNRPD3*, *SNRPD3*, *UPB1*
14	49,550,001	49,650,000	0.335205	*GGT5*
14	49,200,001	49,300,000	0.334916	*RAB36*
14	49,580,001	49,680,000	0.333836	*LOC100520275*, *SUSD2*
14	49,270,001	49,370,000	0.33244	*SPECC1L*
14	49,590,001	49,690,000	0.324805	*CABIN1*
13	50,510,001	50,610,000	0.323567	*FRMD4B*
1	38,460,001	38,560,000	0.322458	*NKAIN2*
13	199,680,001	199,780,000	0.32011	*LOC110256483*
8	6,040,001	6,140,000	0.311989	*LOC110262054*, *LYAR*, *OTOP1*, *TMEM128*, *ZBTB49*, *DRD5*
8	6,090,001	6,190,000	0.311252	*DRD5*

^1^ Represents the start and end of the selection signal regions, and autosome positions are expressed in bp units; ^2^ represents the F_ST_ value between the groups with different NTV; ^3^ represents the genes overlapping with the selection signal regions.

**Table 3:** The partial QTL and genes overlapping with selection signal regions.

SSC	QTL Name	QTL Start (bp) ^1^	QTL End (bp)	Gene Name
2	Bone mineral content	0	13,341,832	*LOC110259708*, *BEST1*, *FTH1*, *RAB3IL1*, *FADS3*, *INCENP*, *C2H11orf24*, *CHKA*, *KMT5B*, *LOC102162815*, *LOC100738812*, *LRP5*, *LOC110259247*, *FADS2*, *LOC106509334*
2	Lumbar vertebra number	21,047,891	146,185,081	*LOC106509513*, *LOC102164448*, *PAM*, *YTHDC2*, *SLCO4C1*, *LOC110255325*, *LOC102158973*, *LOC110259457*, *PPIC*, *SNX24*, *C2H11orf91*, *CD59*, *FBXO3*, *KIAA1549L*, *LOC106509385*
6	Thoracic vertebra number	158,835,021	158,835,025	*LRP8* ^2^
7	Cervical vertebra length	28,939,911	38,532,223	*DEF6*, *TCP11*, *ZNF76*, *SCUBE3*, *PPARD*
7	Cannon bone circumference	31,235,547	31,235,551	*PPARD*
7	Number of ribs	68,061,952	77,142,053	*LOC100736765*, *MYH7*, *NGDN*, *ZFHX2*
7	Thoracic vertebra number	104,557,779	104,557,783	*FOXN3* ^2^
8	Spinal curvature	6,099,724	6,099,728	*LYAR*
10	Vertebra number	13,546,750	69,196,799	*CELF2*, *LOC110255590*, *LOC106505197*, *USP6NL*, *LOC110255605*, *LOC110255591*, *ARMC3*, *LOC106505172*
12	Thoracic vertebra number	0	50,491,372	*BPTF*, *C12H17orf58*, *LOC110256159*, *KPNA2*, *TRNAR-CCG*, *HELZ*, *CA10*
13	Vertebra number	75,687,809	169,726,013	*CMSS1*, *FILIP1L*, *LOC100524713*
14	Cervical vertebra length	62,245,247	81,061,212	*LOC110256649*, *LOC110256651*, *PHYHIPL*, *FAM13C*
16	Number of ribs	28,477,997	34,396,617	*LOC110257315*, *ARL15*, *NDUFS4*

^1^ Represents the start and end of the QTL, and autosome positions are expressed in bp units; ^2^ represents genes located near these QTL.

## Data Availability

Our resequencing data generated in this study have already been uploaded to the NCBI BioProject (https://www.ncbi.nlm.nih.gov/, accessed on 1 January 2023). The accession number is PRJNA 1009598, and a total of 19 raw sequencing data of Licha black pigs were contained in this dataset.

## Supplementary Materials

The following supporting information can be downloaded at https://www.mdpi.com/article/10.3390/genes15040477/s1, Table S1. Summary of sequencing and mapping statistics; Table S2. Information of identified selection signal regions; Table S3. Genes overlapping with selection signal regions; Table S4. Gene ontology (GO) analysis of genes overlapping with selection signal regions (*p*-value < 0.05); Table S5. Pathway analysis of genes overlapping with selection signal regions (*p*-value < 0.05); Table S6. QTL included in or partially overlapped with selection signal regions.
